# Alternating Wetting and Drying Irrigation During Grain Filling Period Improves the Eating Quality and 2AP Content of Aromatic Rice

**DOI:** 10.3390/plants15182890

**Published:** 2026-09-21

**Authors:** Zhengwei Zhang, Qian Li, Guang Li, Le Chen, Xiaobing Xie, Yongjun Zeng

**Affiliations:** 1 Ministry of Education and Jiangxi Key Laboratory of Crop Physiology, Ecology and Genetic Breeding, Jiangxi Agricultural University, Nanchang 330045, China; 15551551951@163.com (Z.Z.); liguang202202@163.com (G.L.);; 2 College of Chemistry and Food Science, Nanchang Normal University, Nanchang 330032, China

**Keywords:** aromatic rice, water management, 2-acetyl-1-pyrroline, starch pasting properties, antioxidant capacity, endogenous hormones

## Abstract

Water management during grain filling plays a critical role in determining rice quality, but its coordinated regulation of eating and aroma quality in aromatic rice remains unclear. In this study, two indica cultivars, Nongxiang 32 and Yuzhenxiang, were subjected to three irrigation regimes: continuous flooding (CF), alternate wetting and drying (AWD), and saturated soil irrigation (SSI). Our results showed that 2-acetyl-1-pyrroline (2AP) content exhibited the highest level under AWD and the lowest under SSI. Starch pasting properties were improved under AWD and SSI, as reflected by higher peak viscosity and breakdown and lower setback values. Further analysis of Nongxiang 32 showed that AWD enhanced amylopectin and total starch contents, elevated proline and putrescine levels, and differentially regulated enzyme activities, maintaining higher proline dehydrogenase activity at early filling stage and favorable Δ^1^-pyrroline-5-carboxylate synthetase, ornithine δ-aminotransferase, and diamine oxidase activities at late filling. Meanwhile, AWD was associated with reduced γ-aminobutyric acid accumulation, consistent with greater precursor availability for 2AP biosynthesis. Antioxidant enzyme activities followed the order AWD > SSI > CF, whereas malondialdehyde and free fatty acids showed SSI > AWD > CF. Hormonal responses differed: gibberellic acid (SSI > AWD > CF), abscisic acid (AWD > SSI > CF), and indole-3-acetic acid (AWD > CF > SSI). Collectively, in Nongxiang 32, AWD optimally coordinates precursor metabolism, enzyme activities, antioxidant defense, and hormone regulation, thus improving both the aroma and starch quality of aromatic rice. This work provides a mechanistic understanding of water-driven quality formation in aromatic rice and offers a feasible irrigation strategy for high-quality aromatic rice cultivation.

## 1. Introduction

Rice (*Oryza sativa* L.) is the staple food for more than half of the world’s population, and its comprehensive quality directly determines market competitiveness and consumer preference [1]. Among rice varieties, aromatic rice holds a premium market position due to its unique fragrance and superior palatability [2]. Grain quality formation in aromatic rice is a complex physiological and biochemical process governed by genotype, environmental conditions, and agronomic practices. The grain-filling stage is particularly critical, as external factors during this period profoundly influence starch biosynthesis, aroma compound accumulation, and overall metabolic homeostasis in developing kernels [3].

2-Acetyl-1-pyrroline (2AP) is the key aroma compound that determines the fragrance quality of aromatic rice. Its biosynthesis is supported by two independent metabolic routes for Δ^1^-pyrroline supply: the glutamate-proline pathway and the polyamine-degradation pathway. Cumulative evidence demonstrates that 2AP biosynthesis in aromatic rice is markedly modulated by agronomic practices, among which water management acts as a critical environmental regulator for grain 2AP accumulation [4]. Moderate water stress has been shown to promote 2AP accumulation in aromatic rice, primarily through enhanced conversion of putrescine (Put) to Δ^1^-pyrroline under low soil moisture [5]. However, the effectiveness of such stress may be stage-dependent, as drought during early grain filling can simultaneously increase 2AP while reducing yield [6]. Alternate wetting and drying has been reported to enhance the activities of proline dehydrogenase (ProDH), Δ^1^-pyrroline-5-carboxylate synthetase (P5CS), and diamine oxidase (DAO); elevate endogenous proline abundance; and consequently accelerate 2AP production [7]. Despite these advances, most studies have focused on binary comparisons (e.g., drying vs. flooding), leaving the relative contributions of a broader spectrum of water regimes—particularly across different grain-filling stages—largely unexplored.

Apart from aroma quality, water regime also governs the starch structure and eating quality of milled rice. Alternate wetting and drying irrigation improves rhizosphere aeration and root vitality, sustains high activity of starch-synthesizing enzymes in the mid-to-late filling stage, elevates amylopectin proportion, and accordingly increases peak viscosity (PV) and breakdown (BD) values while reducing setback (SB) value [8,9]. In contrast, continuous flooding exacerbates soil hypoxia, restricts root respiration, inhibits the accumulation of carbohydrates, elevates the amylose-to-amylopectin ratio, and potentially compromises the final eating quality [10,11]. However, such adverse impacts are not universal: continuous flooding may exert favorable effects on specific quality traits in certain cultivars [12]. In particular, whether sustained saturated soil irrigation differs from alternate wetting and drying in its regulatory effects on starch properties and 2AP accumulation has not been systematically examined.

Phytohormones and antioxidant defense systems are key physiological mediators linking water regimes to grain quality formation. Abscisic acid (ABA) enhances 2AP biosynthesis by promoting proline accumulation and upregulating 2AP synthesis-related genes [13], while also regulating starch biosynthesis in a dose-dependent manner during grain filling [14,15]. Meanwhile, exogenous ABA application enhances antioxidant enzyme activities and alleviates oxidative damage in aromatic rice under stress conditions [16]. Indole-3-acetic acid (IAA) promotes grain filling by extending filling duration and enhancing nitrogen assimilation, which may support 2AP precursor supply [17,18]. Gibberellic acid (GA_3_), in contrast, plays a distinct role: GA generally promotes vegetative growth and α-amylase activity [19,20]. Additionally, exogenous IAA and GA_3_ supplied through seaweed sap application can promote rice growth, nutrient uptake, and yield [21], indicating that hormonal pathways are responsive to external inputs. Similarly, Zn supplementation has been shown to simultaneously increase 2AP content and antioxidant capacity, suggesting a coordinated physiological link between stress defense and aroma formation [22]. These observations suggest that coordinated regulation of hormonal balance and antioxidant capacity may represent a key mechanism linking water management to the concurrent modulation of 2AP accumulation and starch quality.

While individual studies have separately investigated 2AP biosynthesis or starch quality under various water regimes, the integrated regulatory network linking these two quality dimensions—particularly the roles of antioxidant homeostasis and hormonal balance in coordinating their simultaneous modulation—has not been systematically elucidated. Moreover, whether the observed effects are pathway-specific or stage-dependent remains unclear, as most previous work has relied on endpoint measurements rather than time-series analyses throughout grain filling. In this study, three irrigation treatments were imposed throughout the grain-filling period: continuous flooding irrigation, alternate wetting and drying irrigation, and saturated soil irrigation. We systematically quantified the dynamic responses of grain 2AP concentration, starch pasting properties, starch synthetic flux, precursors and rate-limiting enzymes for 2AP biosynthesis, antioxidant capacity, and endogenous phytohormone levels under different water managements. The objective of this study was to elucidate the physiological mechanisms by which water management coordinately regulates aroma formation and eating quality in aromatic rice, and to provide a theoretical foundation and practical guidance for developing green, efficient, and high-quality irrigation strategies for aromatic rice production.

## 2. Materials and Methods

### 2.1. Materials and Treatments

Two conventional non-glutinous indica aromatic rice varieties, Nongxiang 32 and Yuzhenxiang, were selected as test materials. The field trial was conducted in 2023 at the Shanggao Science and Technology Courtyard Experimental Base, Jiangxi Agricultural University. During the grain-filling stage, three water regimes were applied: (1) continuous flooding (CF), in which a 3 ± 0.5 cm standing water layer was maintained, keeping the soil permanently saturated at a soil water potential (SWP) near 0 kPa; (2) alternate wetting and drying (AWD), in which plots were irrigated to a 3 cm standing water layer and then allowed to dry naturally until no standing water remained for 3 days, after which irrigation was resumed, with SWP at the 15–20 cm root zone depth declining to approximately −15 to −25 kPa before re-irrigation; and (3) saturated soil irrigation (SSI), in which a 0 ± 0.5 cm water layer was maintained, with SWP at the 15–20 cm root zone depth generally fluctuating between approximately 0 and −5 kPa. Soil water potential was monitored using tensiometers (TEN-100) installed at the 15–20 cm root zone depth. A completely randomized block design was used with three biological replicates, and individual plot size was 36 m^2^ (11 m × 3.3 m).

On 24 June 2023, seeds were soaked, surface-sterilized, and germinated, and then sown on 26 June. On 22 July, seedlings were manually transplanted at a spacing of 25 cm × 18 cm, with four seedlings per hill. The designated water management treatments were initiated from the beginning of heading and continued until one week before harvest. Grain sampling commenced at 20 days after heading (DAH); panicles were excised, and grains were immediately placed into sealed bags, frozen in liquid nitrogen, transported to the laboratory, and stored at −80 °C for subsequent analysis. Sampling was performed at 5-day intervals until final harvest.

### 2.2. Measurement of 2AP Content

The content of 2AP was measured according to the method described by Xie et al. [23], with minor modifications. Frozen grain samples were equilibrated to room temperature in sealed bags. Then, 2.5 g of paddy rice was weighed into a headspace vial and analyzed for 2AP content using an Agilent 8860-5977B GC-MS system (Agilent Technologies, Beijing, China). Chromatographic separation was performed on a RESTEK Rxi-5ms capillary column (29.5 m × 0.32 mm × 0.25 μm) using the following oven temperature program: 40 °C held for 5 min, ramped at 2 °C min^−1^ to 130 °C and held for 5 min, ramped at 5 °C min^−1^ to 180 °C and held for 2 min, and then ramped at 30 °C min^−1^ to 250 °C and held for 5 min. The inlet temperature was 260 °C, with constant-pressure splitless injection. Mass spectrometric detection employed an electron impact ion source at 200 °C with 70 eV ionization energy, an interface temperature of 250 °C, and a quadrupole temperature of 150 °C. Selected-ion monitoring targeted *m*/*z* 41, 42, 43, 44, 58, 83, and 111. Quantification was performed using an external standard curve prepared with 2AP; the limit of detection and limit of quantification were 5 ng g^−1^ and 20 ng g^−1^, respectively.

### 2.3. Measurement of Metabolite Contents and Enzyme Activities

The contents of proline, malondialdehyde (MDA), free fatty acid (FFA), γ-aminobutyric acid (GABA), amylose, amylopectin, and protein, as well as the activities of starch branching enzyme (SBE), Δ^1^-pyrroline-5-carboxylate synthetase (P5CS), proline dehydrogenase (ProDH), ornithine aminotransferase (OAT), diamine oxidase (DAO), superoxide dismutase (SOD), peroxidase (POD), and catalase (CAT), were determined using corresponding assay kits from Suzhou Grace Biotechnology Co., Ltd. (Suzhou, China). Specifically, the assay principles for ProDH, P5CS, OAT, DAO, and GABA were based on the methods described by Bao et al. [7], and those for MDA, proline, SOD, POD, and CAT were based on the methods described by Ashraf et al. [24]; however, all measurements were performed using commercial assay kits following the manufacturer’s instructions. Amylose and amylopectin contents were measured using the amylose and total starch assay kit as described by Yan et al. [25]. FFA content was determined using the FFA assay kit as described by Song et al. [26]. The contents of Put, IAA, GA_3_, and ABA were determined by Suzhou Grace Biotechnology Co., Ltd. using liquid chromatography, following the methods described previously for Put by Hayat et al. [27], for IAA and ABA by Nonhebel and Griffin [28], and for GA_3_ by Hu et al. [29].

### 2.4. Measurement of Starch Pasting Properties

Starch pasting properties were determined according to the method of Xiong et al. [9]. After harvest, empty and shriveled grains were removed, and the remaining grains were equilibrated at room temperature for 3 months. Then, 3 g of polished rice flour passing through a 100-mesh sieve was weighed into an aluminum can containing 25 mL of double-distilled water and transferred to a Rapid Visco Analyser (RVA TecMaster, PerkinElmer, Inc., Shelton, CT, USA) for starch pasting profile measurement. The parameters included PV, trough viscosity (TV), final viscosity (FV), BD = PV − TV, SB (SB = FV − PV), and pasting temperature (PT).

### 2.5. Data Analysis

All assays were performed in three replicates, and data are presented as means ± standard errors. Graphs were generated using OriginPro 2024 (OriginLab Corporation, Northampton, MA, USA). Statistical analyses were performed using JMP Pro 18 (SAS Institute Inc., Cary, NC, USA) with Tukey–Kramer multiple comparisons. Different letters indicate significant differences (*p* < 0.05).

## 3. Results

### 3.1. 2AP Content in Nongxiang 32 and Yuzhenxiang Grains Under Different Water Management Regimes

**Figure 1:**
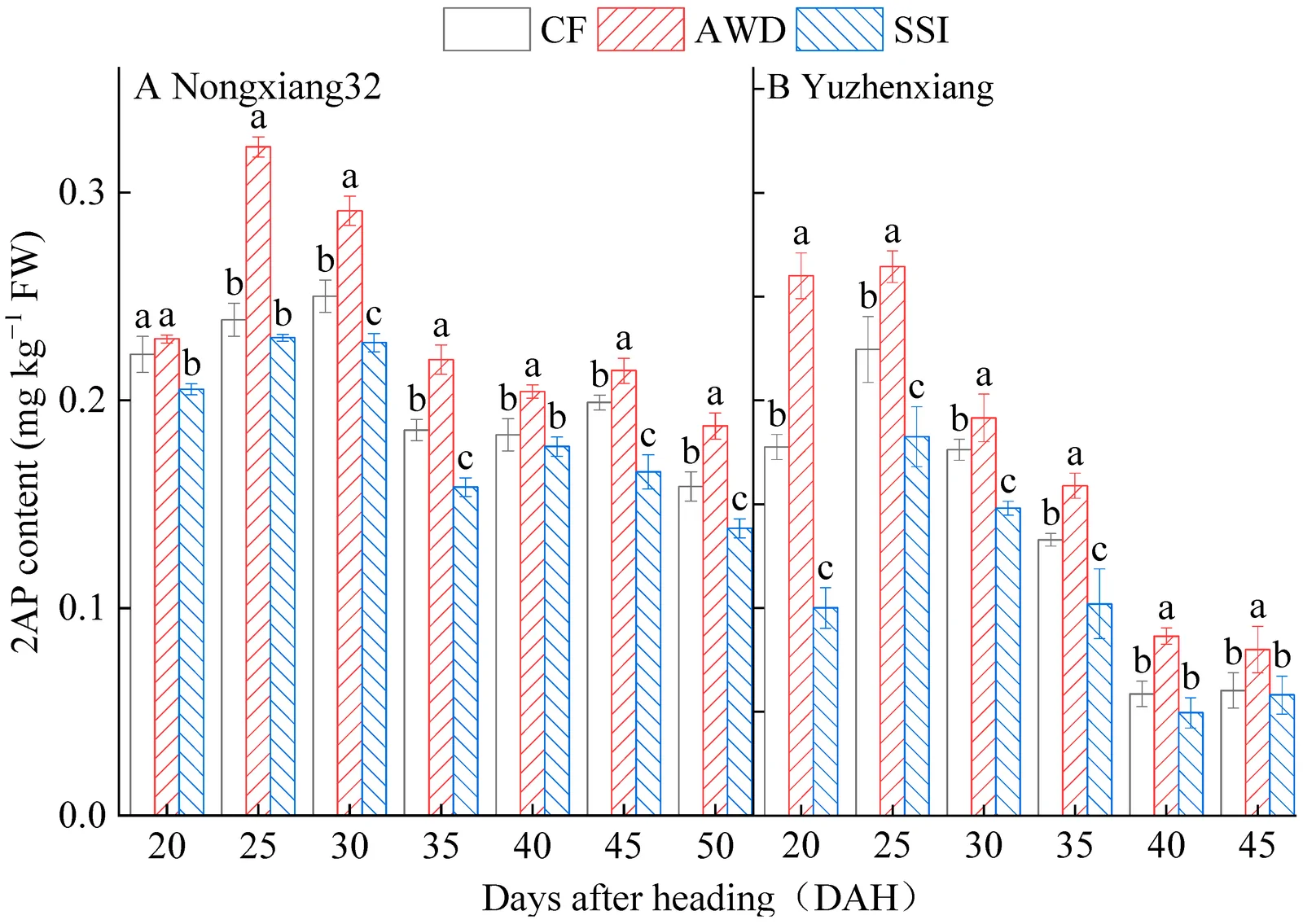
The 2AP content in grains of Nongxiang 32 (**A**) and Yuzhenxiang (**B**) as affected by different water management treatments during grain filling. CF, continuous flooding; AWD, alternate wetting and drying; SSI, saturated soil irrigation; 2AP, 2-acetyl-1-pyrroline. Data are presented as means ± standard errors (*n* = 3). Different lowercase letters above bars indicate significant differences among treatments at the same sampling time point based on the Tukey–Kramer multiple comparison test (*p* < 0.05). FW, fresh weight.

As shown in Figure 1A,B, 2AP content in both aromatic rice varieties initially increased under all water treatments, reaching a peak at approximately 25 DAH and then declining. Throughout the grain-filling period, 2AP content consistently followed the order AWD > CF > SSI. For Nongxiang 32, from 20 to 25 DAH, 2AP accumulated rapidly under all treatments, with the most pronounced increase observed under AWD. For Yuzhenxiang, however, 2AP content under AWD was already significantly higher than under CF and SSI as early as 20 DAH, indicating an earlier response to AWD treatment in this variety. Across both varieties, SSI consistently exhibited the lowest 2AP content throughout the sampling period.

These findings indicated that AWD was most conducive to 2AP accumulation in aromatic rice grains.

### 3.2. Starch Pasting Properties of Milled Rice from Nongxiang 32 and Yuzhenxiang Under Different Water Management Regimes

Water management significantly affected the starch pasting properties of both aromatic rice varieties (Table 1). For Nongxiang 32, AWD and SSI significantly increased PV, BD, and FV, while significantly decreasing SB compared with CF, with no significant differences in TV or PT among the three treatments. For Yuzhenxiang, similar trends were observed for PV, BD, and SB; however, TV under CF was significantly higher than under AWD and SSI. PT remained unaffected by water treatments in both varieties. Notably, no significant differences were detected between AWD and SSI for any of the measured parameters across both cultivars.

These results indicated that AWD and SSI improved starch pasting properties, as reflected by higher PV and BD values and lower SB values, which were generally associated with superior cooking and eating quality.

Given that Nongxiang 32 and Yuzhenxiang showed comparable 2AP contents and starch pasting properties under the three treatments, Nongxiang 32 was selected as the representative cultivar for subsequent physiological and biochemical analyses.

### 3.3. Starch Components, SBE Activity, and Protein Content in Nongxiang 32 Grains Under Different Water Management Regimes

**Figure 2:**
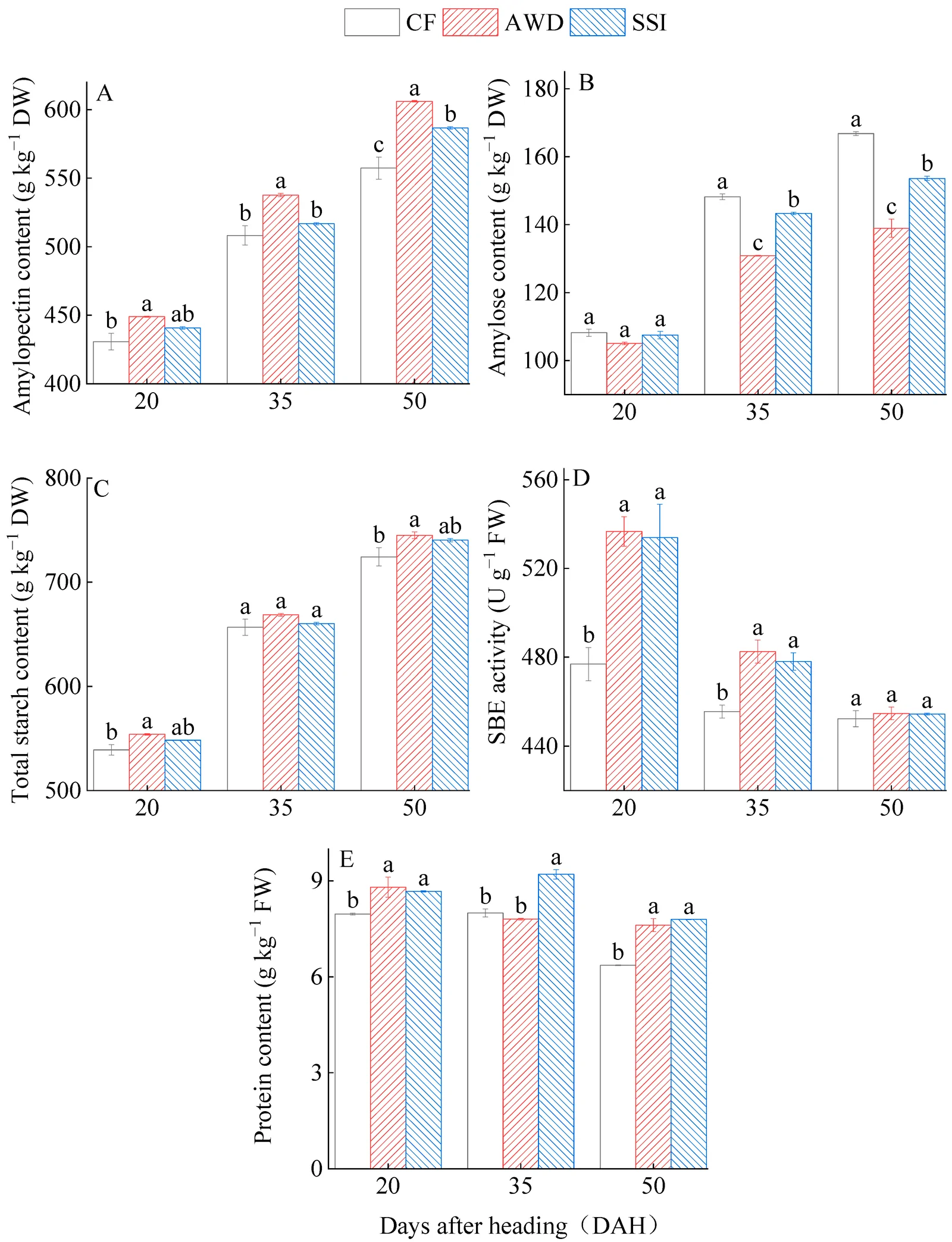
Starch composition, SBE activity, and protein content in Nongxiang 32 grains as affected by different water management treatments during grain filling. (**A**) Amylopectin content; (**B**) amylose content; (**C**) total starch content; (**D**) SBE activity; (**E**) protein content. CF, continuous flooding; AWD, alternate wetting and drying; SSI, saturated soil irrigation; SBE, starch branching enzyme. Data are presented as means ± standard errors (*n* = 3). Different lowercase letters above bars indicate significant differences among treatments at the same sampling time point based on the Tukey–Kramer multiple comparison test (*p* < 0.05). DW, dry weight; FW, fresh weight.

As shown in Figure 2A–C, the contents of amylopectin, amylose, and total starch in Nongxiang 32 grains increased significantly as grain filling progressed. AWD treatment promoted amylopectin accumulation and total starch compared with CF and SSI, while CF promoted amylose synthesis. SBE activity showed a continuous decreasing trend during grain filling; AWD and SSI significantly elevated SBE activity at 20 and 35 DAH compared with CF, whereas no significant difference was observed among the three treatments at 50 DAH (Figure 2D). Protein content in Nongxiang 32 grains slightly decreased with grain ripening (Figure 2E). AWD and SSI treatments obtained significantly higher protein content than CF at 20 and 50 DAH, whereas at 35 DAH, SSI was significantly higher than both CF and AWD.

Overall, AWD promoted greater accumulation of amylopectin and total starch compared with CF and SSI, indicating its superiority in improving starch-related quality traits.

### 3.4. Contents of 2AP Synthesis-Related Precursors, GABA, and Activities of Key Synthetic Enzymes in Nongxiang 32 Grains Under Different Water Management Regimes

**Figure 3:**
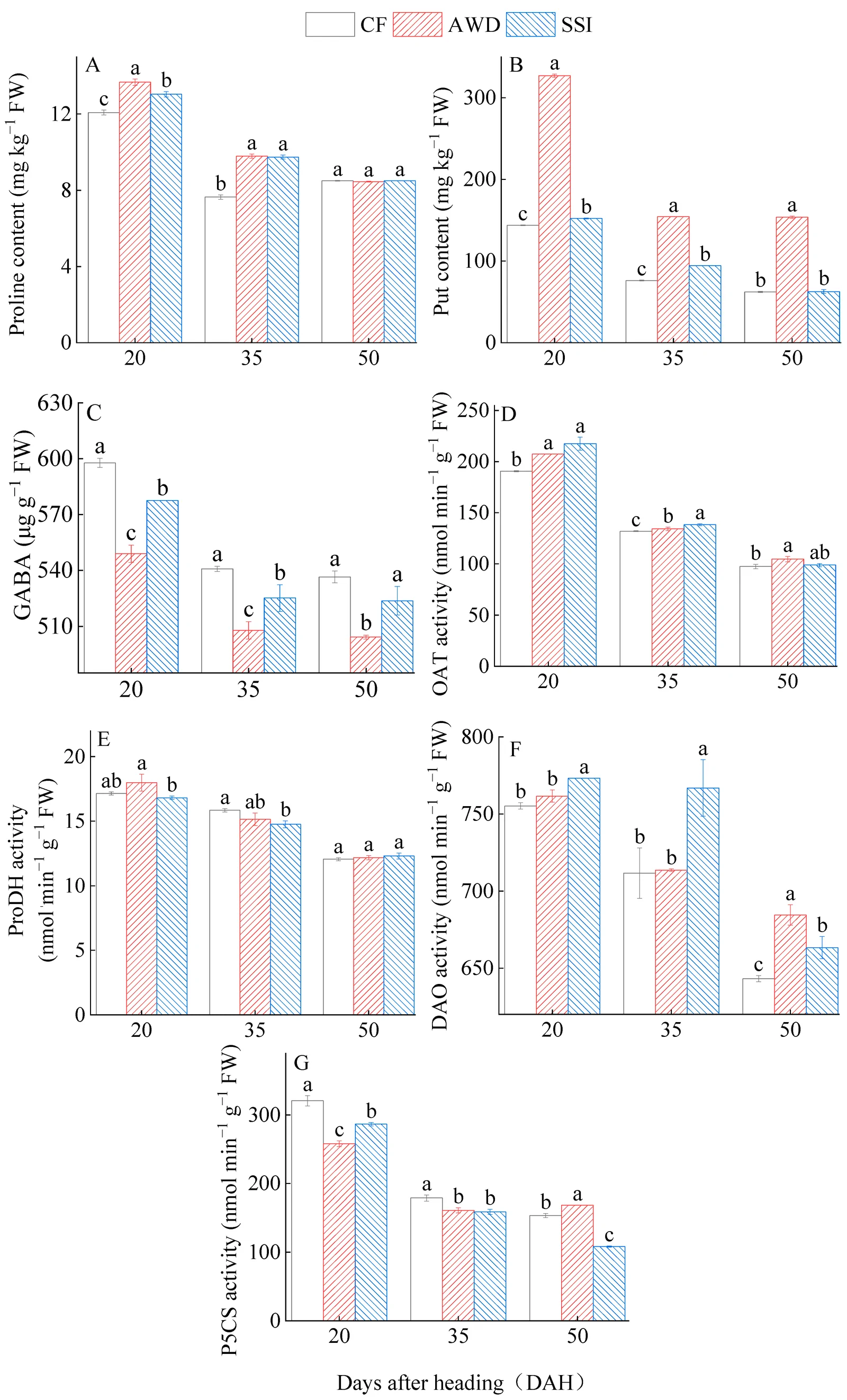
Contents of 2AP-synthesis-related precursors and the competitive metabolite GABA, as well as activities of key biosynthetic enzymes, in Nongxiang 32 grains as affected by different water management treatments during grain filling. (**A**) Proline content; (**B**) putrescine (Put) content; (**C**) γ-aminobutyric acid (GABA) content; (**D**) ornithine δ-aminotransferase (OAT) activity; (**E**) proline dehydrogenase (ProDH) activity; (**F**) diamine oxidase (DAO) activity; (**G**) Δ^1^-pyrroline-5-carboxylate synthetase (P5CS) activity. Data are presented as means ± standard errors (*n* = 3). CF, continuous flooding; AWD, alternate wetting and drying; SSI, saturated soil irrigation; 2AP, 2-acetyl-1-pyrroline. Different lowercase letters above bars indicate significant differences among treatments at the same sampling time point based on the Tukey–Kramer multiple comparison test (*p* < 0.05). FW, fresh weight.

As shown in Figure 3A, proline concentration gradually declined as grain filling progressed. At 20 DAH, AWD treatment showed a significantly higher proline concentration than all other groups, followed by SSI, while CF recorded the lowest value. No significant difference was detected between AWD and SSI at 35 and 50 DAH, both of which were markedly higher than CF at 35 DAH. Put content showed a similar declining trend with grain ripening (Figure 3B). AWD treatment consistently produced the maximum Put content across all three sampling stages; SSI was significantly lower than AWD but higher than CF at each time point, indicating that AWD strongly promoted endogenous Put accumulation in Nongxiang 32 grains. GABA content in Nongxiang 32 grains declined gradually from 20 to 50 DAH (Figure 3C). Across all sampling stages, CF maintained the highest GABA concentration, AWD showed significantly the lowest level, and SSI was intermediate. At 50 DAH, CF and SSI did not differ significantly and both were markedly higher than AWD.

The activities of OAT, ProDH, DAO, and P5CS continuously decreased with the advancement of grain filling (Figure 3D–G). As shown in Figure 3D, SSI showed the highest OAT activity at 20 DAH, followed by AWD and CF. At 35 DAH, the activity ranked in the order of SSI > AWD > CF with significant differences, while only AWD was significantly higher than CF at 50 DAH. As shown in Figure 3E, in contrast to CF, ProDH activity under SSI remained consistently lower throughout the grain-filling period, whereas AWD exhibited a fluctuating pattern relative to CF, being higher than CF at 20 DAH but lower at 35 DAH, with no significant difference between the two treatments at 50 DAH. This temporal shift may be attributable to differential proline turnover kinetics under continuous versus intermittent hypoxia. The stable, slowly replenished proline pool under CF favors a progressive up-regulation of proline catabolism, peaking at the middle filling stage, whereas the repeated wetting–drying cycles under AWD cause the proline pool to fluctuate, driving ProDH activity to oscillate around the CF level. No significant differences in ProDH activity were observed among the three treatments at the late grain-filling stage. DAO activity peaked at 20 DAH for AWD and SSI treatments, with SSI presenting the maximum activity at 35 DAH (Figure 3F). At 50 DAH, AWD maintained significantly higher DAO activity than CF and SSI. In general, SSI elevated DAO activity in the early and middle filling stages, whereas AWD maintained higher activity in the late filling stage. At 20 DAH, P5CS activity was CF > SSI > AWD with significant differences (Figure 3G). At 35 DAH, AWD and SSI had similar activity and were significantly lower than CF. At 50 DAH, AWD treatment recorded the highest P5CS activity, followed by CF, and SSI showed the minimum P5CS activity.

Collectively, water management treatments significantly regulated proline and Put accumulation, as well as key enzyme activities for 2AP biosynthesis, in Nongxiang 32 grains. AWD enhanced proline and Put substrate accumulation, retained higher ProDH activity at early grain filling stage, and maintained favorable P5CS and DAO activities at the late filling stage. In contrast, GABA accumulation was consistently lower under AWD compared with CF and SSI, suggesting that less carbon flux was diverted toward GABA formation under this treatment. Such coordination between substrates and enzymes is consistent with enhanced precursor supply for 2AP formation.

### 3.5. Antioxidant Capacity of Nongxiang 32 Grains Under Different Water Management Regimes

**Figure 4:**
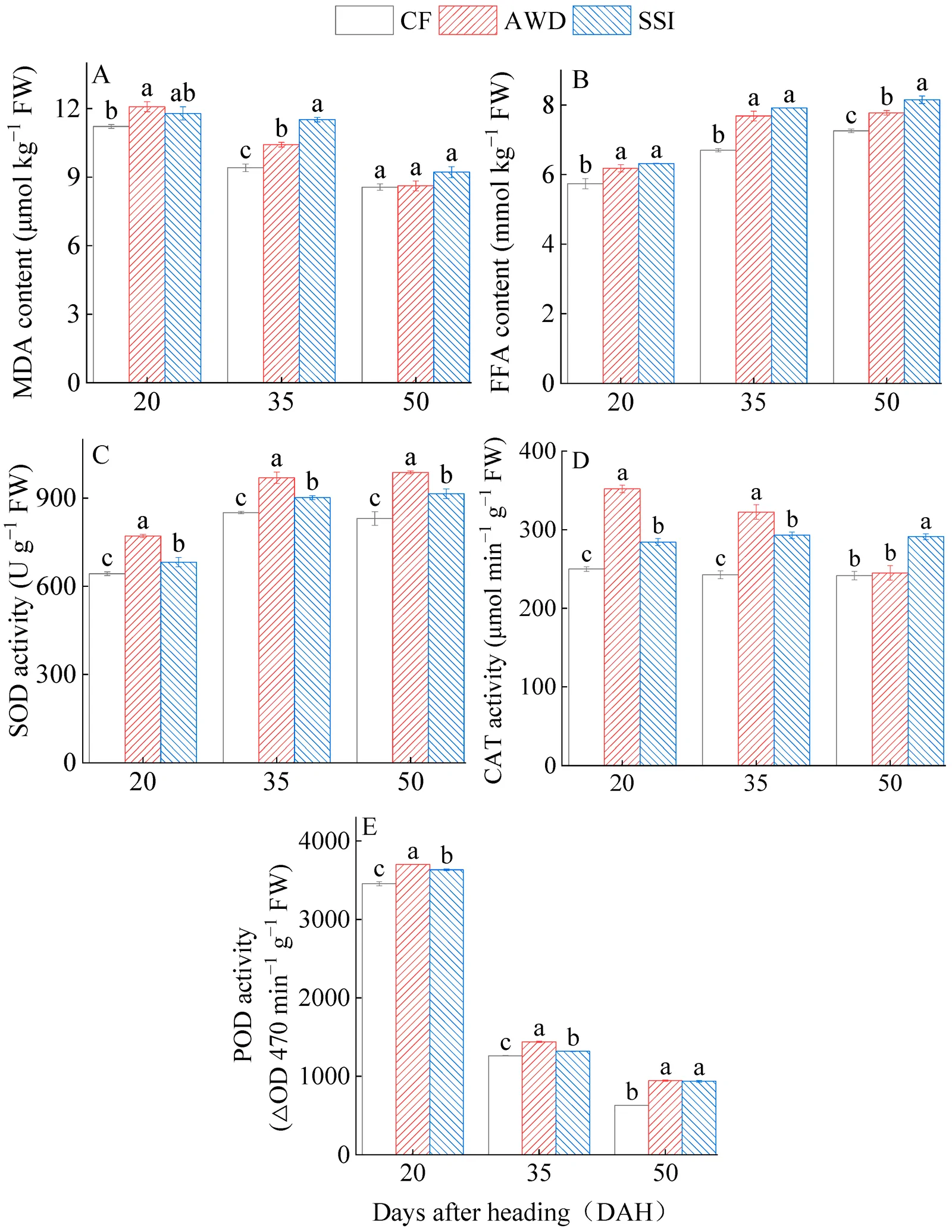
Antioxidant capacity in Nongxiang 32 grains as affected by different water management treatments during grain filling. (**A**) Malondialdehyde (MDA) content; (**B**) free fatty acid (FFA) content; (**C**) superoxide dismutase (SOD) activity; (**D**) catalase (CAT) activity; (**E**) peroxidase (POD) activity. Data are presented as means ± standard errors (*n* = 3). CF, continuous flooding; AWD, alternate wetting and drying; SSI, saturated soil irrigation. Different lowercase letters above bars indicate significant differences among treatments at the same sampling time point based on the Tukey–Kramer multiple comparison test (*p* < 0.05). FW, fresh weight.

MDA content generally decreased as DAH increased (Figure 4A). At 20 DAH, AWD exhibited significantly higher MDA content than CF, whereas no significant difference was detected between AWD and SSI. At 35 DAH, MDA content ranked SSI > AWD > CF, with significant differences across treatments. No significant difference in MDA content was found among the three treatments at 50 DAH. FFA content in Nongxiang 32 grains showed an increasing trend over the grain-filling period (Figure 4B). At 20 and 35 DAH, AWD and SSI treatments significantly elevated FFA levels compared to CF. However, by 50 DAH, SSI showed the highest FFA content, which was significantly greater than that of AWD and CF. Overall, SSI treatment consistently resulted in the highest accumulation of FFAs throughout grain filling.

SOD activity in Nongxiang 32 grains exhibited a general upward trend as DAH progressed (Figure 4C). Significant differences were observed among water management treatments across all three stages. Notably, AWD consistently showed the highest SOD activity, followed by SSI, while CF showed the lowest levels throughout the grain-filling period. As shown in Figure 4D, at 20 and 35 DAH, CAT activity followed AWD > SSI > CF with significant differences among treatments. At 50 DAH, SSI showed significantly higher CAT activity than CF and AWD. POD activity decreased continuously with the increase of DAH (Figure 4E). At 20 and 35 DAH, POD activity followed the order AWD > SSI > CF with significant differences among treatments. By the final stage (50 DAH), both AWD and SSI maintained similarly higher activities than CF, which dropped to the lowest level.

Collectively, AWD exhibited the best overall antioxidant performance, maintaining the highest SOD, POD, and CAT activities across most of the grain-filling period, followed by SSI, while CF showed the lowest enzyme activities. Although both AWD and SSI triggered higher antioxidant enzyme activities than CF, AWD achieved a more favorable balance between ROS scavenging and oxidative damage, as evidenced by its lower MDA and FFA contents compared with SSI.

### 3.6. Endogenous Hormone Levels in Nongxiang 32 Grains Under Different Water Management Regimes

**Figure 5:**
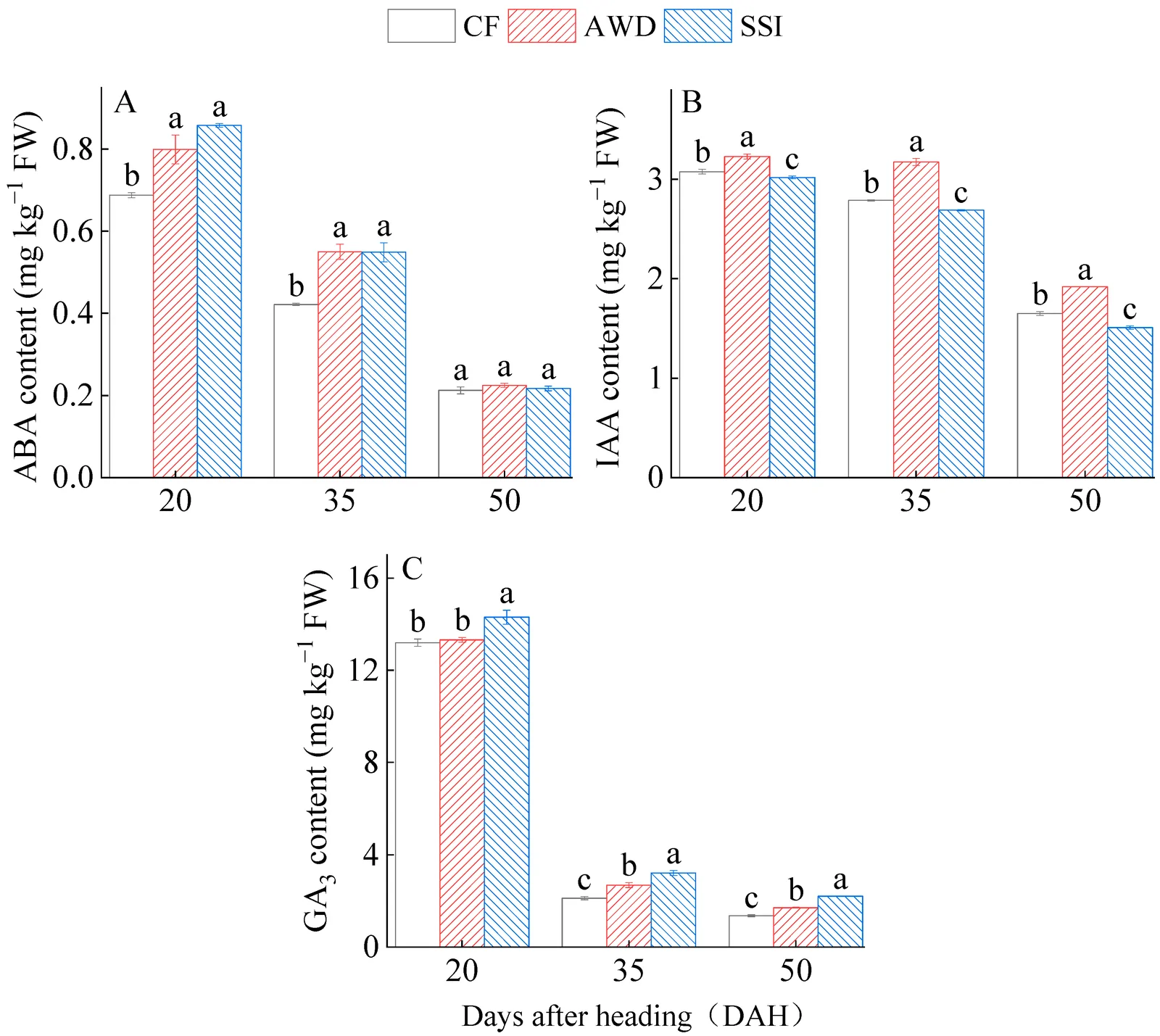
Endogenous hormone levels in Nongxiang 32 grains as affected by different water management treatments during grain filling. (**A**) Abscisic acid (ABA) content; (**B**) indole-3-acetic acid (IAA) content; (**C**) gibberellic acid (GA_3_) content. Data are presented as means ± standard errors (*n* = 3). CF, continuous flooding; AWD, alternate wetting and drying; SSI, saturated soil irrigation. Different lowercase letters above bars indicate significant differences among treatments at the same sampling time point based on the Tukey–Kramer multiple comparison test (*p* < 0.05). FW, fresh weight.

As shown in Figure 5A–C, the contents of ABA, IAA, and GA_3_ in Nongxiang 32 grains generally decreased as DAH increased. At 20 and 35 DAH, ABA levels under AWD and SSI were significantly higher than those under CF (Figure 5A). However, by 50 DAH, no significant differences were observed among the three treatments, all of which dropped to low levels. IAA content followed the order AWD > CF > SSI with significant differences among treatments across all stages (Figure 5B). GA_3_ content showed a sharp decline after 20 DAH (Figure 5C). At 20 DAH, AWD and SSI significantly increased GA_3_ levels compared with CF. By 35 and 50 DAH, GA_3_ content followed the order SSI > AWD > CF, with significant differences observed among all treatments.

Collectively, these results indicated that AWD and SSI maintained relatively higher ABA levels throughout grain filling. Notably, AWD was most effective in enhancing IAA content, while SSI exhibited the highest GA_3_ level at the grain-filling stage.

## 4. Discussion

Water management during grain filling profoundly influences the quality formation of aromatic rice [30]. Although it is widely recognized as a critical regulator, the integrated physiological mechanisms through which water regimes coordinately modulate 2AP biosynthesis, starch metabolism, antioxidant homeostasis, and hormonal balance remain inadequately understood. Nongxiang 32 was selected for in-depth mechanistic dissection based on its comparable 2AP and starch pasting profiles with another variety (Yuzhenxiang); however, because phenotypic similarity does not guarantee equivalent physiological mechanisms, all mechanistic measurements were performed exclusively on Nongxiang 32, and the mechanistic conclusions below are restricted to this cultivar. Our results revealed that AWD proved to be the most effective strategy, significantly promoting 2AP accumulation and improving starch pasting quality, while maintaining favorable redox homeostasis and hormonal balance. Collectively, these findings advance the mechanistic understanding of water-driven quality formation in aromatic rice and provide a practical theoretical framework for optimizing irrigation strategies in high-quality aromatic rice production.

### 4.1. AWD Optimizes 2AP Accumulation Through Coordinated Regulation of Precursors, GABA Competition, and Key Enzymes

Water management during grain filling plays a pivotal role in the accumulation of 2AP, the key aroma compound in aromatic rice. In this study, AWD consistently produced the highest 2AP content throughout grain filling for both Nongxiang 32 and Yuzhenxiang, whereas SSI exhibited the lowest 2AP level (Figure 1A,B). These results indicated that periodic drying–rewetting cycles, rather than CF or SSI conditions, provided the most favorable environment for 2AP biosynthesis in aromatic rice. Previous studies have shown that moderate soil water stress can induce 2AP accumulation [5,31]; however, our findings further suggest that intermittent stress under AWD is more effective than persistent mild stress under SSI.

The accumulation of specific precursors plays a decisive role in 2AP formation [2]. Put serves as a key substrate in the polyamine pathway, providing pyrroline intermediates for 2AP synthesis via DAO catalysis [32]. The glutamate-proline pathway supplies P5C for 2AP biosynthesis through three key enzymes: P5CS and OAT convert glutamate and ornithine into P5C, respectively, while ProDH generates P5C via the oxidation of proline [33,34]. In addition to precursor supply, the competitive partitioning of shared metabolic intermediates also influences 2AP formation. GABA is a metabolite that branches off from the polyamine-degradation pathway at the level of γ-aminobutyraldehyde (GABald), a common intermediate shared with 2AP biosynthesis [35]. The partitioning of GABald between GABA formation and 2AP synthesis may directly influence the efficiency of 2AP accumulation [36]. The synergistic interplay between precursor pools and their corresponding catalytic enzymes is essential for sustaining 2AP production. For example, exogenous proline treatment significantly increased the contents of proline, P5C, and Δ^1^-pyrroline in aromatic rice grains, thereby promoting 2AP biosynthesis and improving certain grain quality traits [37]. The responses of key enzymes involved in 2AP biosynthesis to water management were stage-specific rather than uniform. ProDH activity was highest under AWD only at 20 DAH, whereas CF showed the highest activity at 35 DAH, and no treatment difference occurred at 50 DAH (Figure 3E). At 50 DAH, OAT, DAO, and P5CS were higher under AWD than under CF and SSI (Figure 3D,F,G). Thus, AWD did not sustain all 2AP-biosynthesis-related enzyme activities throughout grain filling; instead, it appeared to coordinate a stage-specific pattern—higher ProDH at the early stage and higher OAT, DAO, and P5CS at the late stage—thereby extending the window for precursor conversion and 2AP biosynthesis. Moreover, the lower GABA content under AWD is consistent with the possibility that a greater proportion of GABald was available for 2AP synthesis rather than being consumed in GABA production; however, this interpretation remains correlative because GABald and metabolic flux were not directly measured. The prolonged high DAO, OAT, and P5CS activities under AWD extended the biosynthesis window, whereas the transient early surges under SSI and CF limited precursor mobilization. The drying and rewetting cycles under AWD likely supplied repetitive mild stress signals that both elevated precursor levels and sustained enzyme activities. Collectively, in Nongxiang 32, the association between higher 2AP content under AWD and the concurrent patterns of precursor abundance, enzyme activity, and lower GABA accumulation suggests that AWD may support 2AP biosynthesis through sustained precursor supply and reduced competition from the GABA branch, underscoring the importance of extending the biosynthetic window under periodic water stress. It should be noted that glutamate decarboxylase activity was not measured, and our interpretation of GABA as a competing sink for GABald is based on the inverse relationship between GABA and 2AP contents rather than on direct flux quantification. GABA can be synthesized via both the GAD-mediated glutamate pathway and the polyamine degradation pathway, and only the latter shares GABald with 2AP biosynthesis. Future work combining GAD activity assay with stable-isotope labeling will be needed to directly quantify carbon partitioning between these pathways.

### 4.2. AWD and SSI Modulate Starch Pasting Properties via SBE-Mediated Regulation of Starch Composition

Starch pasting properties are key determinants of rice palatability and cooking quality. Higher PV and BD values generally indicate better palatability, whereas lower SB reflects reduced retrogradation [38,39,40]. In this study, both AWD and SSI significantly increased PV and BD while decreasing SB compared with CF, with no significant differences between the two non-flooded treatments (Table 1). Pasting temperature remained unaffected, suggesting that water management primarily modulates starch swelling and retrogradation rather than gelatinization onset. The improved pasting profiles were closely associated with starch compositional changes. AWD and SSI promoted amylopectin and total starch accumulation while reducing amylose content (Figure 2A–C), resulting in a higher amylopectin-to-amylose ratio—a shift known to enhance PV and BD and suppress SB [39,41]. Consistently, AWD and SSI showed significantly higher SBE activity than CF at both 20 and 35 DAH (Figure 2D). Since SBE catalyzes amylopectin branching, the elevated SBE activity is consistent with a higher degree of amylopectin branching, although this inference rests on enzyme activity and pasting parameters rather than direct chain-length measurements. Confirming such structural changes will require HPAEC-PAD profiling of the amylopectin chain-length distribution in future work.

Notably, despite similar pasting properties between AWD and SSI, AWD accumulated significantly more total starch and amylopectin than SSI (Figure 2C). This apparent discrepancy suggests that pasting quality is governed more by amylopectin structure and the amylopectin-to-amylose ratio than by absolute starch content. The enhanced starch accumulation under AWD may be attributable to a rehydration-induced compensatory response that facilitated assimilate partitioning toward grains [42,43]. In contrast, CF, with lower SBE activity under prolonged reducing conditions, led to higher amylose content, which intensifies hydrogen bonding and accelerates retrogradation, resulting in lower BD, higher SB, firmer texture, and greater cooling-induced retrogradation [44]. Despite elevated protein levels under AWD and SSI compared with CF (Figure 2E), the overall pasting quality remained superior, driven by enhanced amylopectin branching and reduced SB. Collectively, in Nongxiang 32, AWD and SSI improved starch pasting quality through SBE-mediated regulation of amylopectin structure, with AWD showing additional benefits in starch accumulation.

### 4.3. Synergistic Regulation of Grain Quality by Water-Management-Mediated Antioxidant and Hormonal Balance Systems

Water deficit typically triggers reactive oxygen species (ROS) accumulation but also activates enzymatic antioxidant systems to counteract oxidative stress [45,46]. In this study, AWD and SSI exhibited significantly higher MDA and FFA contents than CF (Figure 4A,B), confirming that non-flooded treatments induced oxidative stress. However, compared with SSI, AWD maintained higher SOD, POD, and CAT activities for most of the grain-filling period, accompanied by lower MDA and FFA levels (Figure 4). These results indicated that although both treatments triggered oxidative stress, AWD possessed a superior ROS scavenging capacity and more effectively alleviated lipid peroxidation. The enhanced antioxidant performance under AWD is consistent with previous observations that methyl jasmonate [47] or GABA [48] can similarly elevate SOD, POD, and CAT activities in aromatic rice grains, accompanied by a significant increase in grain 2AP content and rice quality. Mechanistically, however, AWD operates through a physical wetting and drying cycle that endogenously primes antioxidant defenses during the drying phase and permits recovery upon rewetting—a dynamic “priming–repair” rhythm fundamentally distinct from the sustained chemical stimulus applied in exogenous treatments [49]. This periodic stress-recovery pattern likely confers a more sustainable redox balance than persistent stress under SSI or transient chemical induction, as reflected by the consistently lower MDA and FFA levels under AWD. Collectively, these findings suggest that intermittent mild stress, rather than continuous stress or exogenous elicitors, is more effective in coordinating long-term antioxidant defense and aroma formation.

Phytohormones serve as key signals linking environmental cues to physiological responses. IAA functions as a central signaling hub that integrates abiotic stress perception and developmental reprogramming, governing stress-responsive gene expression, tissue adaptive remodeling, and extensive signaling crosstalk with ABA, GA_3_, and other phytohormones under fluctuating environmental conditions [50]. IAA promotes photoassimilate transport from source to sink and sustains grain filling by facilitating starch biosynthesis [17,51]. In this study, IAA content under AWD was significantly higher than under CF and SSI (Figure 5B). Accordingly, the elevated IAA level in AWD likely contributed to the higher total starch and amylopectin contents observed in this treatment. ABA, a central stress hormone, accumulates under water deficit, inducing stomatal closure and activating stress-responsive genes [52], while also regulating starch synthase activities and 2AP synthesis [13,53]. AWD and SSI significantly increased grain ABA content (Figure 5A), consistent with stress responses. Notably, the concurrent elevation of IAA and ABA under AWD may establish a synergistic coordination, as supported by recent evidence in rice. Teng et al. [54] demonstrated that ABA accumulation under moderate soil drying promotes IAA biosynthesis through the T6P-OsTAR2 pathway and downregulates auxin signaling repressors (*OsIAA29*, *OsIAA24*), thereby enhancing starch synthesis and grain filling in inferior spikelets. According to the results of our study, ABA may promote storage carbohydrate accumulation and osmotic regulator (proline and Put) synthesis, while IAA regulates cell expansion and grain filling, ensuring nitrogen transport to grains for 2AP precursor supply. We therefore hypothesize that the co-elevation of ABA and IAA under AWD reflects a coordinated growth–stress balance favoring 2AP synthesis and amylopectin accumulation, although direct evidence from hormone-response gene expression or genetic manipulation is required to confirm this causality.

In contrast, GA_3_ content was highest under SSI, followed by AWD. GA generally promotes vegetative growth and α-amylase activity [19,20], and exogenous GA application has been shown to significantly reduce grain 2AP content [55]. The elevated GA_3_ under SSI may therefore have enhanced α-amylase-mediated starch degradation, reducing starch accumulation and diverting carbon skeletons and ATP away from 2AP biosynthesis—partly accounting for the lower 2AP and total starch contents under SSI. Conversely, the relatively low GA_3_ under AWD likely favored stable carbohydrate accumulation, conserved energy for secondary metabolism, and maintained adequate substrate availability for 2AP synthesis. A recent work in Arabidopsis roots shows that IAA signaling can reduce bioactive GA by inducing GA2OX6 and GA2OX8, the key GA-deactivating enzymes [56]. In our study, AWD maintained the highest IAA while keeping GA_3_ at the lowest level (Figure 5B–C). We therefore speculate that a similar relationship may operate under AWD, whereby elevated IAA signaling could contribute to restraining GA_3_ accumulation. If so, the combination of high IAA and low GA_3_ would be expected to act in a coordinated manner to favor grain quality: elevated IAA would support assimilate transport and grain filling, while low GA_3_ would limit α-amylase-mediated starch degradation, thereby jointly preserving starch accumulation and maintaining carbon skeletons and precursor availability for 2AP biosynthesis—consistent with the higher starch, amylopectin, and 2AP contents observed under AWD (Figure 1 and Figure 2A–C).

In summary, in Nongxiang 32, AWD outperformed CF and SSI by achieving an optimal integration of antioxidant defense and hormonal regulation. Under AWD, enhanced ROS scavenging alleviated oxidative damage, while favorable IAA and ABA levels promoted starch accumulation and 2AP biosynthesis, and reduced GA_3_ prevented excessive starch catabolism. These coordinated physiological adjustments collectively underpin the superior grain quality and aroma formation observed under AWD. It should be acknowledged that the hormonal changes observed here were correlated with, rather than demonstrated to cause, the changes in starch and 2AP traits. Nevertheless, the direction of hormonal changes under AWD—higher IAA and ABA, lower GA_3_—is consistent with established mechanisms by which these hormones regulate starch accumulation and 2AP biosynthesis, although this inference does not exclude the possibility that both are parallel consequences of the same water-stress signal. Causality would require verification through exogenous hormone application or genetic manipulation.

In this study, all enzyme, metabolite, and hormone assays were performed on bulk grain tissue, which may mask cell-type-specific and spatially heterogeneous responses to alternating wetting–drying cycles. The rice caryopsis contains functionally distinct tissues (aleurone, subaleurone, starchy endosperm, and maternal tissues) that differ in metabolic activity and stress perception, so the precursor, enzyme, antioxidant, and hormone signals reported here represent tissue-averaged responses. Recent advances in single-cell and spatial transcriptomics offer a means to resolve such cell-type-specific stress responses in plants [57]. Future work adopting these approaches will help refine and validate our proposed mechanistic model of 2AP and starch coordination during grain filling. Additionally, all enzyme activities were measured at the catalytic level, restricting our interpretation to associations among enzyme activity, substrate pools, and metabolites. ProDH and DAO may be predominantly substrate-driven, as their activity changes paralleled the proline and Put pools. The activity changes of P5CS and SBE, by contrast, may involve transcriptional or post-translational regulation: both are key enzymes developmentally regulated during grain filling, and their sustained elevation at specific stages under AWD may reflect regulation at the transcriptional or post-translational level; however, as gene expression and protein abundance were not measured in this study, this remains speculative and unverified. The fluctuating redox environment under wetting–drying cycles may also affect enzyme activity through post-translational modification. Thus, the proposed model rests solely on enzyme activity, substrate, and metabolite data, and the regulatory hierarchy remains to be clarified through transcript profiling, protein quantification, and substrate-feeding or inhibitor experiments.

## 5. Conclusions

In conclusion, AWD increased 2AP accumulation in the aromatic rice cultivars Nongxiang 32 and Yuzhenxiang. In-depth mechanistic analyses conducted in Nongxiang 32 revealed that the promotion of 2AP biosynthesis by AWD is related to precursor supply, key enzyme activities, and a possible competition from the GABA branch. AWD also improved starch pasting properties via increased SBE activity, alleviated oxidative damage through enhanced antioxidant enzyme activities, and maintained a favorable hormonal balance with elevated IAA and ABA but reduced GA_3_. In contrast, SSI caused excessive oxidative stress, whereas CF led to higher amylose content and inferior eating quality. Collectively, our findings in Nongxiang 32 reveal the integrated regulatory network by which AWD simultaneously modulates aroma and starch quality through coordinated precursor metabolism, enzymatic dynamics, antioxidant defense, and hormonal balance. AWD is therefore recommended as a promising irrigation strategy for improving the eating and aroma quality of aromatic rice. However, its water-saving and yield benefits remain to be verified in future studies that quantify irrigation water use and grain yield.

## Figures and Tables

**Table 1:** Starch pasting properties of milled rice from Nongxiang 32 and Yuzhenxiang under different water management regimes during grain filling.

Cultivar	Water Management	PV (cP)	TV (cP)	BD (cP)	FV (cP)	SB (cP)	PT (℃)
Nongxiang 32	CF	2652 ± 7 ^b^	1478 ± 7 ^a^	1174 ± 5 ^b^	2560 ± 8 ^b^	−92 ± 7 ^a^	74.71 ± 0.02 ^a^
	AWD	2754 ± 9 ^a^	1499 ± 7 ^a^	1247 ± 14 ^a^	2588 ± 7 ^a^	−165 ± 12 ^b^	74.59 ± 0.11 ^a^
	SSI	2731 ± 6 ^a^	1496 ± 9 ^a^	1235 ± 10 ^a^	2569 ± 10 ^ab^	−162 ± 9 ^b^	74.73 ± 0.15 ^a^
	*F*-test	***	ns	***	*	***	ns
Yuzhenxiang	CF	2744 ± 4 ^b^	1506 ± 11 ^a^	1238 ± 13 ^b^	2575 ± 10 ^a^	−169 ± 12 ^a^	75.53 ± 0.02 ^a^
	AWD	2778 ± 6 ^a^	1456 ± 11 ^b^	1322 ± 13 ^a^	2533 ± 10 ^b^	−245 ± 10 ^b^	75.52 ± 0.02 ^a^
	SSI	2790 ± 8 ^a^	1465 ± 6 ^b^	1326 ± 12 ^a^	2547 ± 6 ^ab^	−243 ± 12 ^b^	75.52 ± 0.01 ^a^
	*F*-test	***	**	***	**	***	ns

Values are means ± standard errors (*n* = 3). Different superscript letters within the same column indicate significant differences among treatments (*p* < 0.05). CF, continuous flooding; AWD, alternate wetting and drying; SSI, saturated soil irrigation; PV, peak viscosity; TV, trough viscosity; BD, breakdown (BD = PV − TV); FV, final viscosity; SB, setback (SB = FV − PV); PT, pasting temperature. *, **, and *** indicate significant differences at *p* < 0.05, *p* < 0.01, and *p* < 0.001, respectively; ns, not significant.

## Data Availability

The data that support the findings of this study are available from the corresponding author upon reasonable request.
